# The Relationship Exploration between Public Migration Attention and Population Migration from a Perspective of Search Query

**DOI:** 10.3390/ijerph17072388

**Published:** 2020-04-01

**Authors:** Chun Li, Jianhua He, Xingwu Duan

**Affiliations:** 1 Institute of International Rivers and Eco-Security, Yunnan University, Kunming 650091, Yunnan, China; lichun@ynu.edu.cn; 2 School of Resources and Environment Science, Wuhan University, Wuhan 430079, Hubei, China; hjianh@126.com

**Keywords:** population migration, search query, Baidu Index, urban agglomeration

## Abstract

Rapid population migration has been viewed as a critical factor impacting urban network construction and regional sustainable development. The supervision and analysis of population migration are necessary for guiding the optimal allocation of urban resources and for attaining the high efficiency development of region. Currently, the explorations of population migration are often restricted by the limitation of data. In the information era, search engines widely collect public attention, implying potential individual actions, and freely provide open, timelier, and large-scope search query data for helping explore regional phenomena and problems. In this paper, we endeavor to explore the possibility of adopting such data to depict population migration. Based on the search query from Baidu search engine, three migration attention indexes (MAIs) are constructed to capture public migration attention in cyber space. Taking three major urban agglomerations in China as case study, we conduct the correlation analysis among the cyber MAIs and population migration in geographical space. Results have shown that external-MAI and local-MAI can positively reflect the population migration inner regions and across regions from a holistic lens and that intercity-MAI can be a helpful supplement for the delineation of specific population flow. Along with the accumulation of cyber search query data, its potential in exploring population migration can be further reinforced.

## 1. Introduction

Along with the rise of a city network, which is constructed under the push of different kinds of urban elements flows, the interactions among different cities have been emphasized in the planning of urban areas, including the interaction of population, material, information, technique, etc. Hereinto, population interaction or population migration is one of the most important aspects. The floating of population is not only the flowing of individual human but also the transfer of demand, information, and technique carried by individuals [1,2]. They discriminately impact economic, social, and political development of both resettled areas and out-migrating areas [3,4]. Timely measuring and analyzing of population migration are particularly crucial for suitably planning urban space and distributing urban resources.

Related explorations on population migration have been concerned as hotspots since the 1990s. A larger body of researches have been conducted, such as the labor market performance, social and physical status of migration [5,6,7], the causes of migration flow [8,9,10], the consequent impacts of migration [11,12,13], the changing migration policies [14,15,16], the classification research of population migration [17,18], the spatial pattern of population migration [19], etc. These researches have been conducted mainly based on three kinds of data: national censuses data, regional field survey data, and cyber big data. In the traditional migration researches, population censuses and field survey are the principal sources to provide population data [20,21]. For instance, Zhu [22] explored the determined factors in urban area which influence migrants’ settlement intention based on the data from a survey on the floating population in the coastal area of Fujian Province. He et al. [23] adopted national census data to examine the distinctive spatial patterns of floating and Hukou population and evaluated their consequent impact on Chinese urbanization and industrialization. With the development of cyber space and the popularization of personal mobile termination, numerous researches have implemented under the assistance of data from cyber space exploration of the change, characteristic, and pattern of population migration [24,25,26,27]. For instance, Blumenstock [28] analyzed migration pattern based on mobile phone records and revealed more subtle patterns that were not detected in the government population survey. Zagheni et al. [29] used geolocated data for about 500,000 users of the social network website “Twitter” to predict turning points in migration trends and to improve the understanding of migrant populations.

Those researches have contributed largely to promoting the understanding of the progress of population migration and their impact. However, the deficiencies in migration data still exist. Studies based on national censuses data can explore the migrants in a large range but with a relatively large time interval of ten years, which hinders the short time-series analysis of population migration, and little can be inferred for specific years between censuses and for recent trends [29]. The researches based on field survey can provide detailed migration information, but it asks for a lot of time, manpower, and material resource to deploy, which are expensive for many researches. Simultaneously, the field survey often has a certain spatial location and cannot cover a large spatial scope. The increasing cyber data has opened up a new opportunity to deepen our understanding of population migration. However, studies based on the network big data always need to deal with extensive data and complicated procedures in acquiring and processing the data. At the same time, some data sources are not available openly, such as cellphone signal data and GPS data of resident activities, because those types of data include much individual private information that is protected by national law. A type of data with open, timelier, and easy-taking characteristics is necessary for effectively investigating the migration population.

With the growing application of search engine in cyber space, search query data has been brought out to reflect the preference of public attention, which is generated from the personal behavior of Internet search. This kind of data with opening and timelier characteristic has provided effective support for analyzing regional phenomena and problems [30,31,32,33]. In such context, the concern is triggered about its applicability in population migration research. In current information era, most people tend to take migration after an inquiry of destinations. Web search engine as the most widely used Internet tools provides massive information to the migrants and obtains relevant public attention on the specific subject of migration. The relationship between Internet search query data and population migration deserves more attention. However, the relationship between them is still unclear and there are a number of questions to be raised: can the search query data generated from migration-related information search offer some clues about population migration? If they can, how are they related? Do cites with higher cyber search quantity have a larger migration population than the cities with lower search quantity?

Based on these questions, this paper endeavors to answer them and to propose a new angle to analyze population migration. A hypothesis can be made that the search queries generated from individual migration-related search can positively reflect population migration. Based on the search query data from Baidu search engine, we construct a series of migration attention indexes (MAIs) to explore public attention on migration. Taking three main urban agglomeration areas of China as study area, the correlation analysis has been utilized to explore the relationship between MAIs and population migration to test the hypothesis. This paper is organized as follows. Section 2 introduces the study area and data. Section 3 elucidates the methodology of this paper, including the method and indicators that we applied in this paper. Section 4 reports the result of correlation analysis between MAI and population migration. Section 5 conducts further discussion based on the results in our study area. Last, we conduct the conclusion of this paper.

## 2. Study Area and Data

### 2.1. Study Area

**Figure 1:**
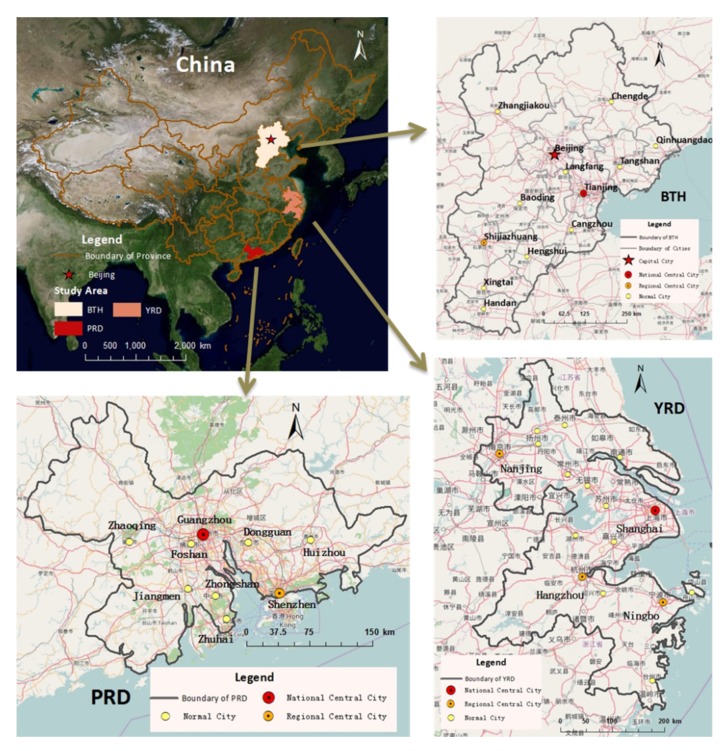
Location of the study area.

To verify the relationship between search query data in cyber space and population migration in geographical space, we select three urban agglomerations in China as case study: Beijing-Tianjin-Hebei metropolitan region (BTH), the Yangtze River Delta (YRD), and the Pearl River Delta (PRD). There are 38 cities located in these regions, 10 cities from BTH, 16 from YRD, and 9 from PRD, as shown in Figure 1. These regions are chosen based on the following reasons: (1) Extensive population migration can be detected in these areas. In 2015, the migration population in these areas has reached more than 8 million in total, accounting for 30.77% in China. Exploration of migration in these regions can avoid the influence of random migration under the support of large quantities. (2) These regions with relatively higher internet penetration offer adequate search query data. By the end of 2015, internet penetrations of core cities in those three urban metropolitan areas are separately 76.5% for Beijing, 73.1% for Shanghai, 78.4% for Guangzhou, and 83.2% for Shenzhen. More widespread application of the internet can be identified in almost all the provinces cover BTH, YRD, and PRD [34]. (3) They are the most significant areas for China’s urban system construction. These areas occupy approximately 5.09% land of China but account for 23.65% and 39.87% of national permanent residents and gross domestic product in 2015. Research on them can offer more information to guide the coordinated development of urban areas in China.

### 2.2. Data

The data used in this paper include the population migration data, search query data, and socioeconomic data. (1) There are three kinds of population migration data used in this study: the net inflow population, intercity population flow, and the floating population. The net inflow population delineates the total population migrated into the city during a specific period. Intercity population flow is the population migrate from the original city to the terminal city. Based on the prevalent use of series Tencent’s applications (e.g., Wechat is the most used software for 79.6% of Chinese netizens), more precise expression on the migration of population in China can be provided by Tencent migration map under the support of enormous user base. Considering the merit of Tencent migration map and avoiding the self-certification of Baidu, we obtained the net inflow population and intercity population flow from Tencent migration map (https://heat.qq.com/qianxi.php) through web crawler technology. Due to the specific Hukou policy in China (which has been regarded as the central mechanism underlying the unsettled nature of the floating population), the floating population has been defined as the population living in the objective city more than six months without local registered Hukou [35]. It was obtained through the deviance calculation of permanent residential population and household population in the local city, which were collected from regional statistical bureaus. (2) We obtain the search query data based on the support of Baidu search engine, which is the most widely used search engine in China and freely provides the search trend of objective terms through Baidu Index (http://index.baidu.com/). The average daily queries of each migration keyword versus the name of the city (e.g., job + Beijing) from 1 January 2015 to 31 December 2015 were collected based on Baidu Index. (3) Relevant socioeconomic data were acquired from regional statistical bureau, including the Tertiary Industrial Output-Value, Participant Rate of Urban Basic Medical Care System, the number of schools, etc. The migration reasons were collected from the dynamic monitoring survey of China’s migration population in 2015, which was conducted by the National Health and Family Planning Commission of China.

## 3. Methodology

**Figure 2:**
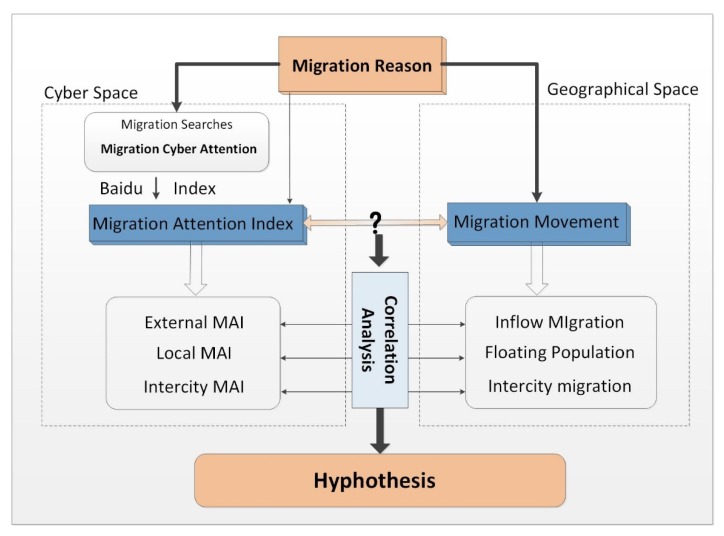
Research framework.

We endeavor to verify the relationship between public attention on migration which was provided by search query in cyber space and the population migration in geographical space. Migration attention indexes (MAIs) are proposed to express public attention on migration comprehensively. Based on the different original location of migration search, we construct three MAIs as local-MAI, external-MAI, and intercity-MAI to delineate the public attention generated from local city, attention from external areas, and attention flow among urban areas; then, the correlation analysis is conducted between MAIs in cyber space and urban migrants in geographical space to further verify the aforementioned hypothesis. The framework of this paper can be illustrated in Figure 2.

Specially, the net inflow population, intercity population flow, and the floating population have been collectively adopted to depict the movement of population in this paper. The definition of migration for them can be separately clarified as follows: The net inflow population of a city is defined as population that the city has received from the external areas, which is the result of movement of people with different origins and the same destination; the intercity population flow is also defined as the movement of people, which happens among different cities; and the floating population of a city is defined under the Hukou policy of China (which has been regarded as the central mechanism underlying the unsettled nature of the floating population), of which the migration can be explained as the change in the place of personal residence.

### 3.1. Migration Attention Index Based on Baidu Search Query

To verify the hypothesis that the migration-related search queries from individual users can positively reflect the population migration, three issues should be concerned: (1) what are the main driving factors cause population migration; (2) how to express those factors in cyber space through search query data; and (3) how to synthesize those search query data to comprehensively express public attention on migration in cyber space. For the first issue, based on the dynamic monitoring survey of China’s migration population in 2015, we have conducted the statistic of population percentage on different migration reasons to confirm the main factors which cause population migration. For the second issue, a series of search keywords expressing different migration reasons has been selected. The Baidu Index of keywords versus the name of city has been collected to reflect the public attention on migration in cyber space. For the third issue, migration attention indexes (MAIs) have been constructed to integrate public attentions generated based on different migration reasons.

#### 3.1.1. Confirmation of Main Migration Driving Forces

To pointedly select search keywords that load public attention on migration. First, we confirm the main reason for population migration based on the dynamic monitoring survey of China’s migration population in 2015. The percentage statistics of migrant population based on diverse migration reasons in the three different urban agglomerations are deployed. The results have been shown in Table 1; we can see that work and trade, that study and training, that accompanying transferring of family members, and that relocation are the main migration factors in the study area. The percentages of population who migrate for the four reasons separately occupy 75.70%, 85.39%, and 89.77% in Beijing-Tianjin-Hebei metropolitan region, the Yangtze River Delta, and the Pearl River Delta.

Due to the transferring of family members always accompanying family relocation [36], we have viewed them as one perspective and marked as relocation. Therefore, three main reasons for population migration have been confirmed as *work and trade*, *study and training*, and *relocation*. 

#### 3.1.2. Selection of Search Keywords from Baidu Index

To better exhibit and exploit search query data, relevant search exploit services based on search query data are produced, typically as Google Trend (www.google.com/trends/) and Baidu Index (http://index.baidu.com/). A series of researches have been conducted to analyze data from Google Trend and Baidu Index; the robustness and effectiveness of them have been assessed [37,38,39]. In China, compared to Google, which is the largest search engine in the world, Baidu shares more internet search engine market. In 2016, there are 731 million netizens in China and the number of search engine users has reached 602 million [34]. Hereinto, Baidu shares 77.07% of the Internet search engine market, which is more than Google China. Especially, Vaughan and Chen [40] collected and compared the data from Google and Baidu and found that Baidu Index can offer more search volume data than Google Trend did in China. Under such context, the Baidu Index is employed in this paper to obtain public search attention in the cyber space.

Focusing on the three main migration reasons, we endeavor to confirm the search keywords which reflect public attention on migration. The confirmation of search keywords is comprehensively confirmed under five steps. First, according to the least effort principle in network information retrieval behaviors, users incline to choice the search keywords in their common language with brief and straightforward features [21,36,41,42,43,44]. We set the candidate keywords with brief structure and expressed them in Chinese. Second, the specific content of candidate keywords was derived from the three main migration reasons. Relevant search terms for them were selected by brainstorming common words used in searching for migration and review of related literature [21,45,46,47]. Third, we have compared the daily average search query data of designated search keywords with similar words during the same period to confirm that the selected keywords are the most popular search keywords in the related aspects. For example, “租房 (house renting)” has been compared to “出租 (rent)” and “租赁 (lease)”; collecting and organizing their average daily Baidu Index can find that “house renting”(11,795) gets much more attention than “rent”(477) and “lease”(636). Fourth, we sift the candidate words to follow the principle of search query data for each keyword in each city to be delineated as a sequential time series with a yearly resolution. Fifth, the correlation analysis between the last candidate keywords has been conducted and the one with a high correlation with others has been removed to reduce data redundancy. Through the comprehensive consideration of keyword selection, the last keywords can be viewed as not only representing the meaning itself but also including some clues for other potential keywords. Finally, six Chinese keywords from Baidu index have been confirmed to express public attention on migration in cyber space as list in Table 2.

#### 3.1.3. Construction of MAIs

The migration attention indexes (MAIs) are designed to comprehensively express public attention on migration in cyber space comprehensively. First, we combine the candidate search keywords with the name of objective cities to obtain the cityward migration keywords, such as “school + Beijing”, “house price + Shanghai”, “recruitment + Shenzhen”, etc.; second, the average daily search volume of these cityward keywords are acquired based on Baidu Index from 1 January 2015 to 31 December 2015; third, the population percentages of different migration reasons are viewed as index weight to synthesize the corresponding Baidu Index into MAIs; fourth, according to the origin location of Baidu Index, the *local-MAI*, *external-MAI*, and *intercity-MAI* are separately constructed to express public migration attention on objective cities from internal area of the objective cities, external areas, and other specific cities. The relationship among those indexes can be depicted as follows:

(1) $$MAI_{i}=External\_MAI_{i}+Local\_MAI_{i}$$

(2) $$External\_MAI_{i}=\underset{j=1}{\sum }intercity\_MAI_{ij}$$

where *i* is the objective city, *j* is the original city, *MAI_i_* is the total migration attention city *i* has achieved from all regions, and *local-MAI_i_* and *External-MAI_i_* are separately the total migration attention city *i* has received from the urban internal area and external areas. *Intercity-MAI_ij_* is the public migration attention derived from city *j* to city *i*. The formula of those indexes can be shown as follows:

(3) $$Local\_MAI_{i}=\overset{3}{\underset{n=1}{\sum }}W_{in}\times BI_{n}/MAI_{max}$$

(4) $$External\_MAI_{i}=\underset{j=1}{\sum }\overset{3}{\underset{n=1}{\sum }}W_{ijn}\times BI_{n}/MAI_{max},i\neq 1$$

(5) $$Intercity\_MAI_{ij}=\overset{3}{\underset{n=1}{\sum }}W_{ijn}\times BI_{n}/MAI_{max},i\neq j$$

where *BI_n_* is the average daily volume of Baidu Index about different search keywords under migration reason *n*; *W_in_* and *W_ijn_* are the weights of *BI_n_*, which are defined by the proportion of people who migrate into city *i* for this reason; and *MAI_max_* is the max absolute value of MAI indicators.

### 3.2. Correlation Analysis between MAIs and Population Migration

#### 3.2.1. Correlation with Urban Migrants

To investigate the relationship between public migration attentions in cyber space and population migration in geographical space, we conduct the correlation analysis between MAIs and urban migrants. In the cyber space, local-MAI, external-MAI, and intercity-MAI were selected to represent public migration attentions with different originations to objective cities; in geographical space, floating population, inflow population, and intercity population flow were collected. Regarding the diverse kinds of migration and different definition of MAIs, the correlation analysis have been conducted from three aspects: (1) the correlation between local-MAI and floating population, which reflects the relationship between migration attention generated from the local city and actual floating population inside the city; (2) the correlation analysis between external-MAI and inflow population, which explores the relationship between migration attentions received from the external areas and actual inflow population of the objective city; and (3) the correlation analysis between intercity-MAI and intercity population flow, which investigates the relationship between cyber migration attention flows and the actual population flows in the geographic space. Pearson correlation coefficient is employed to test such correlations, the formula can be shown as follows:

(6) $$r=\frac{1}{n-1}\overset{n}{\underset{i=1}{\sum }}\left(\frac{X_{i}-\bar{X}}{\delta_{X}}\right)\left(\frac{Y_{i}-\bar{Y}}{\delta_{Y}}\right)$$

where *r* is the correlation coefficient of the two indexes and *n* is the number of cities.

#### 3.2.2. Correlation with Urban Migration Attractiveness

Furthermore, we inquired about the relationship between urban external-MAI in cyber space and urban comprehensive attractiveness for migrants (UAM) in geographical space to further test the validity of the proposed indicators. Based on the push-pull theory which has been widely used in analyzing migration action and willing [48,49,50,51], we confirmed the UAM from urban pull perspective. The major migration reasons confirmed by the dynamic monitoring survey of China’s migration population have been employed as reference in confirming the objective content of UAM, including work and business, study and training, and relocation. The three aspects separately correspond with the three major migration reasons as job opportunity and income level, living condition, and educational opportunity of children. Based on the data availability principle and integrated analysis of previous studies, eight indicators with respect to three aspects of urban pulling power have been selected as shown in Table 3. From job and income perspectives, Tertiary Industrial Output-Value (TIV) [52] and Urban Residents’ Per Capita Disposable Income (IPC) [43] were employed to reflect urban job opportunities and income level; Unemployment Rate (UR), Participant rate of Urban Basic Medical Care System (RBM) [53], and Per Capita Living Area (LPC) [43] were utilized to expose the living condition of local residents; Number of Regular Primary Schools (PSN), Number of Regular Secondary Schools (SSN), and Number of University (UN) were applied to reveal educational opportunity for migrants’ children [44].

Last, we adopted the principal component analysis (PCA) to integrate the index system and to obtain the indicator which reflects urban comprehensive attractiveness for migrants. The components with eigenvalues greater than 1 and the cumulative ratio of total variance greater than 85% are extracted and rotated with the varimax method in SPSS 19.0 (International Business Machines Corporation, New York, USA), so that each factor has the minimum number of high load variables, which can be expressed as follows:

(7) $$UAM_{k}=\overset{m}{\underset{i=1}{\sum }}\left[A_{i}\cdot \overset{n}{\underset{j=1}{\sum }}C_{ij}\times X_{kj}^{*}\right]$$

where *UAM_k_* is urban comprehensive attractiveness for migrants of city *k*; *m* is the number of major components which make the cumulative ratio of the total variance greater than 85%; *A_i_* contributes the major components *i* to UAM of the city; *n* is the number of indexes; *C_ij_* is the contribution of index *j* to the major components *i*; and X^*^_kj_ is the standardized value of index *j* in city *k*.

## 4. Results

### 4.1. Correlation between External-MAI and Urban Inflow Migrants

**Figure 3:**
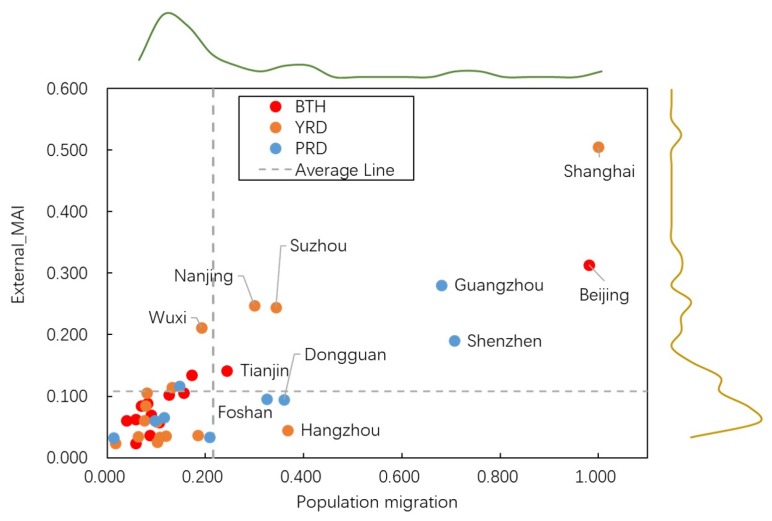
Scatter plot of external-MAI and migration population.

According to the definition of MAI, the migration tendency of the person from the outside areas can be conveyed through external-MAI. Under the assistance of relevant migration data from the Tencent map, we engaged in exploring the relationship between external-MAI and urban migration population. Pearson correlation coefficient was adopted to reveal the relationship between them; the results have been shown in Table 4 and Figure 3. As we could observe, there are significant positive correlations between external-MAI and population migration in the three urban agglomerations. The Pearson coefficients are 0.948, 0.876, and 0.879 separately in BTH, YRD, and PRD, which has a holistic coefficient of 0.844. All of them have passed the significance test at 99% confidence level. Focused on their spatial heterogeneity, the cities of BTH has the highest correlation.

**Figure 4:**
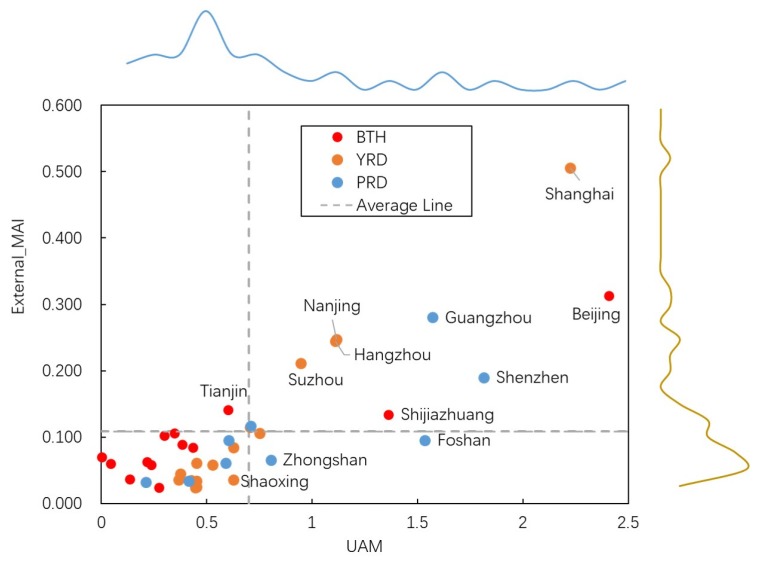
The scatter plot of external-MAI and UAM.

Applying the principal component analysis, we obtained UAM of target cities based on the statistical data; the correlation study was deployed between the comprehensive UAM and external-MAI. As shown in Table 5 and Figure 4, we could observe a significant correlation between the UAM and external-MAI in the study areas. The coefficients of the whole area, BTH, YRD, and PRD are separately 0.829, 0.924, 0.984, and 0.789. The high correlation between them illustrated that urban received external-MAI is highly correlated to the attractiveness of urban itself. The relationship between such a cyber-based index and a traditional statistic-based index can be implied.

Furthermore, the Pearson correlation coefficients between the selected indexes and external-MAI have been calculated, as shown in Table 6. We can see that all the two indexes for job opportunities and income levels have the highest correlation with external-MAI in the study area. For the living condition perspective, a positive correlation can be observed between the Participant Rate of Urban Basic Medical Care System and external-MAI in BTH and YRP. However, significant correlations cannot be observed between the unemployment rate per capita living area with external-MAI. Paying attention to the education opportunities, significant correlations can be found in BTH and YRD between the three educational indexes and population attention index. In PRD, only the number of primary schools significantly correlates with external-MAI. In the three urban agglomerations, the strongest correlations are depicted between the Tertiary Industrial Output-Value and external-MAI, which reflect job opportunities in the areas being conventionally attractive for the potential migrants. Insignificant low correlation between the unemployment rate per capita living area with external-MAI can be detected.

### 4.2. Correlation between Local-MAI and Floating Population Inner City

**Figure 5:**
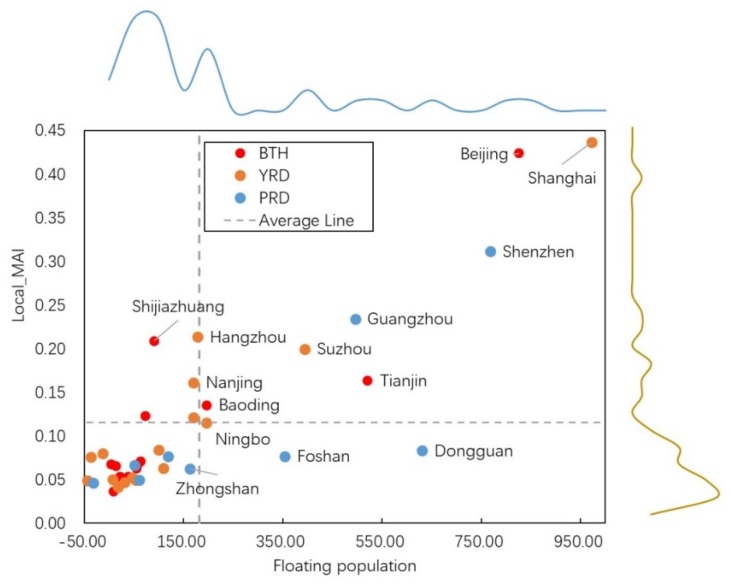
The scatter plot of local-MAI and floating population.

The results of correlation analysis between local-MAI and local floating population have been shown in Table 7 and Figure 5. We can see that, no matter in the whole study area or the individual urban agglomeration, high correlation coefficients were gained. Especially in the YRD, the relevant coefficient has arrived at 0.950. PRD has a relatively lower value but is still higher than 0.75. Divided by the median value of local-MAI and local floating population, the cities in the study area can be divided into four types. Thereinto, 78.95% of them has high-high or low-low features. For the cities with higher-than-average floating population and higher-than-average local-MAI, there are three located in the BTH (Beijing, Tianjin, and Baoding), two in YRD (Shanghai and Suzhou), and two in PRD (Shenzhen and Guangzhou).

**Figure 6:**
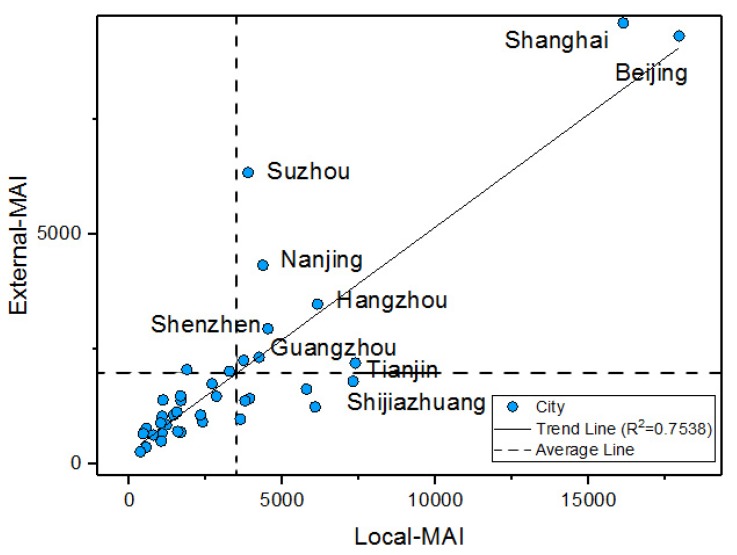
Scatter plot of external-MAI and Local-MAI.

To further excavate information from MAI, the relationship between local-MAI and external-MAI has been explored; the results are shown in Figure 6. There is a highly positive correlation between the two indexes, of which the r is 0.7538 and *p* is 0.01. It is shown that the city with higher local-MAI also has a higher external-MAI

### 4.3. Correlation between Intercity-MAI and Intercity Population Flow 

**Figure 7:**
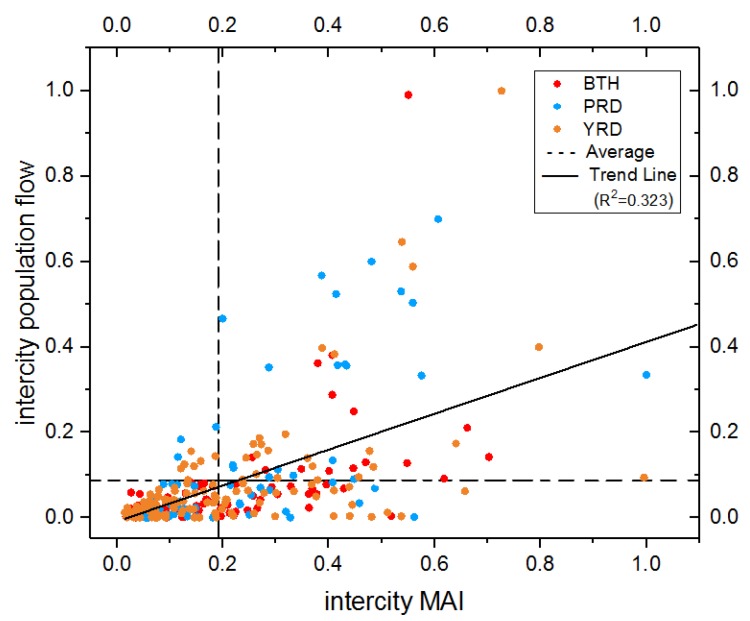
The scatter plot of intercity-MAI and intercity population flow.

To explore the relationship between intercity-MAI and intercity population flow, the results have been shown in Table 8 and Figure 7. As we could notice, the average value of intercity-MAI is 1.00, Guangzhou-Shenzhen has the highest intercity-MAI at 5.19; and Shenzhen-Chengde has the lowest index of 0.04. For the individual urban agglomeration, the intercity-MAI among Beijing, Tianjin, and Shijiazhuang has the highest top three values in BTH. The same level of intercity-MAI can be found in YRD for Shanghai, Hangzhou, and Suzhou. In PRD, such level interactions are observed between Guangzhou, Shenzhen, and Foshan.

Under the correlation analysis of these two indexes, a moderately positive correlation can be observed in the study area (See Table 8). For the three urban agglomerations, PRD has the highest correlation among them and the correlation in BTH and YRD represents a relatively lower level. There are 59 pairs of cities that have a high-high correlation pattern (high intercity-MAI and high intercity population flow), there into 15 pairs in BTH, 24 pairs in YRD, and 20 pairs in PRD; 147 pairs of cities exhibited the low-low correlation pattern, of which, in BTH, YRD, and PRD, are separately 46, 69, and 32. These two kinds of correlation patterns occupy 75% of the total. Although relevant correlation coefficients of intercity-MAI are relatively limited, it can capture the interaction trend of population flow at an acceptable level.

## 5. Discussion

### 5.1. Evaluation of MAIs

The massive population migration is the specific phenomenon and the inevitable driving force promoting the urbanization of population in China and many developing countries. The collection of urban MAIs can obtain the public intention of migration based on individual search actions and can offer exploration of population migration. Depending on the MAIs, we analyzed the correlation relationship between local-MAI and external-MAI; a high correlation has been discovered. It implied that the city with relatively higher local-MAI has a higher external-MAI. Migration may be active in the high-high cities, such as Shanghai, Beijing, and Shenzhen. According to the dynamic monitoring survey of China’s migration population in 2015, the proportions of floating population inside these three cities separately reached 40.26%, 38.02%, and 67.51%, which are much higher than the average value of China at 18.00%. Besides, they have separately occupied 12.22%, 11.99%, and 8.64% (ranked top 3) of the whole inflow population of the three urban agglomerations, which has the most dynamic migration in China. The predominant roles of them in attracting population outside the city are declared. Active migration movement can be detected to support the hypothesis derived from the correlation relationship between local-MAI and external-MAI.

Analyzing the correlation of external-MAI with UAM, the reasonability of external-MAI can be verified through the high correlation with conventional statistics analysis. Based on the push-pull theory of migration, in the cities with higher urban pulling force, more influx of population can be observed. Through the calculation of UAM, which depicted urban pulling force, the city with higher external-MAI can observe higher UAM. The feature of external-MAI coincides with the setting of push-pull theory. Further, exploring the relationship between external-MAI and the indexes which reflect urban migration attractiveness, there are significant correlations between tertiary industrial output-value and urban residents’ per capita disposable income with the external-MAI in the whole study area. Most of the cities with higher job opportunities and income levels receive more migration attentions from the outside area. This finding coincides with the dynamics monitoring survey of migration population suggesting a migration reason in Table 1 (*work and trade* as the predominant migration reason), which can represent the ability of MAI indexes in capturing the impact of migration reasons. With respect to the indexes described urban living conditions, there are no significant correlations between the population migration attention and unemployment rate or per capita living area, this results may correspond to the great exception of potential migrants for their future urban condition, which can be explained by the Todaro migration model from the perspective of development economics. Todaro migration model argues that the migration of population is based on the “expected profit” of migrants. The hardships in urban life have not obtained enough attention from potential migrants, particularly for the rural-urban migrants who lack the necessary information as they enter a new different world [43]. Further, the more schools a city has, the more public migration attention it receives. The positive correlation existing between the education indexes (the number of primary schools, secondary schools, and universities) and external public migration attention exposes that the educational opportunities also intensify the level of urban migration attention. In PRD, the focus of educational concern only derives from the consideration of secondary schools; significant correlation has not been observed between the number of the other two levels of schools in the area, which may be attributed to the relatively lower education level of Guangzhou Province (the administrative province that PRD belongs to) than the other two urban agglomerations.

Besides, we further adopted the neoclassical theory in population migration to explore the reasonability of MAIs. The per capita disposable income of urban residents, which has been viewed as the direct index depicting the possibility for migrants to improve economic benefit, has been adopted to conduct the correlation analysis with external-MAI; the results have shown that the external-MAI has significant positive correlation with the per capita disposable income of urban residents (with the correlation coefficients 0.650, 0.945, 0.752, and 0.780 separately in the whole study area, in BTH, in YRD, and in PRD). The reasonability of MAIs can be further identified.

### 5.2. Relationship with Migrants

With the correlation analysis of external-MAI and urban migration population, we could observe a significantly positive correlation. The resource endowment gap between different urban areas (e.g., economic development level, environmental quality, promotion of opportunities for individuals, etc.) triggers personal develop exception and forges migrant needs in flowing among diverse regions [22,43]. Collecting information about the targeted city by employing the search query engine is an efficient and low-cost approach to supplement requisite information before deploying actual migration to external areas. As noted as the correlation results of external-MAI and urban inflow migrants in the study area, we can accept the hypothesis that the migration-related search queries from individual users were able to positively reflect urban inflow migrants. External-MAI can be applied to reflect urban inflow migrants on the annual scale.

The high correlation between local-MAI and the floating population inside cities was a signal to prove their close relationship. Nowadays, the floating population inner city has become an influential part in enacting urban planning and policy. Generally speaking, the floating population lived with relatively weaker urban amenities than the local population [54,55]. The desire of improving current conditions was more intensive for them, which was delineated by the high demand for new job opportunities, study chance, and the possibility of improving living quality, etc. Driven by such basic needs, the corresponding search query can be brought into the cyber space and raises the high correlation between local-MAI and floating population inside the city.

Intercity migration has already become one of the significant migration models in current China. We analyzed the correlation between intercity-MAI and intercity population flow in 2015; a similar positive correlation can be observed as 0.57 (*p*-value 0.00) in the whole area. The results show that the representativeness of intercity-MAI for population flow between different cities was effective, but the correlation relationship was relatively limited. It might be caused by two main reasons: (1) The selection of search keywords cannot cover every reason for migration flows. A unique keyword system may exhibit some deviation in reflecting the driving force of every population flow interaction; (2) migration movement has a lagging feature. It may happen a few months, years, or a much longer time after the search action. It also may be canceled or indefinitely delayed after information acquisition through searching, which makes the relatively lower correlation between intercity-MAI and intercity population inflow. Generally speaking, the correlation between intercity-MAI and population flow is still on an acceptable level. It can be a supplement for the population flow research of insufficient data. In future work, the construction of a targeted search keywords system for objective population flow can be adopted to remedy such drawbacks.

Besides, for the three MAI indexes, the different correlation coefficients in the three urban agglomerations revealed that regional disparity exists. We calculated the variance (VAR) and coefficient of variation (CV) of relevant correlation coefficients of three MAI indexes, as shown in Table 9. It can be seen that all the VARs are lower than 0.01 and that all the CVs are lower than 10%. The robustness of external-MAI, local-MAI, and intercity-MAI in reflecting population can be partly ensured in the study area. 

Furthermore, we have tested the significance of slope of the three trend lines, which were separately fitted based on external-MAI and inflow population, local-MAI and floating population, and intercity-MAI and population flow to identify whether MAI indexes could steadily reflect the migration situation in different urban agglomerations. All the significances of slopes have been rejected by significant testing at a significant level of 0.05 (sig = 0.43, 0.19, and 0.86 for external-MAI, local-MAI, and intercity-MAI). The null hypothesis could be accepted as there is no significant deviance between the slopes. Although the representations of MAI are diverse in different regions, the deviances are nonsignificant.

## 6. Conclusions

Migration population has immense potential to push urbanization process in current China and other developing countries. Exploration of population migration based on multisource data can bring more information about the noticeable driving force of urban development. In the information and network era, the MAI indexes can reveal how the public put their attention on migration-related items. Based on the cyber-based indexes, we explore the relationship between public migration attention in cyber space and urban migration population in geographical space inner region, across region, and between regions. The results can answer the questions mentioned in the introduction that search query data based MAI indexes can positively reflect the situation of migration population inner region and across region and, for the population flow, that it is an alternative supplement and support when relevant data is deficient.

Population migration is a complex process driven by diverse forces; this paper conducted a series of analyses from the perspective of search query data in cyber space. However, some limitations exist: First, the selection of continuous search keywords is limited by the short period of data acquisition from the search query engine. Following the incremental collection of search query data, more suitable search keywords should be selected to cover different aspects of public migration attention to thus better delineate the difference and characteristic of urban migrant population; second, this paper focus on the panel data analysis; future work will emphasize on the time-series analysis and excavate more information from a dynamic perspective.

## Figures and Tables

**Table 1:** Migration population percentage of different migration reason in the different urban agglomerations.

Reason	Beijing-Tianjin-Hebei	the Yangtze River Delta	The Pearl River Delta
Work and trade	43.61%	58.53%	69.17%
Occupation mobility	4.39%	2.13%	2.31%
Study and training	9.37%	6.73%	5.51%
Accompanying transferring of family members	11.56%	10.37%	11.15%
Join relatives and friends to find a means of living	4.61%	4.25%	2.93%
Relocation	11.16%	9.75%	3.93%
Deponi of Hukou	1.42%	0.67%	0.26%
Marriage	5.78%	3.91%	2.02%
Others	8.09%	3.65%	2.71%

**Table 2:** Selection of search keywords.

Reason	The Chinese Keywords	The English Translation
Work and business	招聘,租房	recruitment, house renting
Study and training	学校	school
Relocation	房价,地图,天气	house price, map, weather

**Table 3:** Indicator system of urban pulling power.

Aspects	Indicators	Unit
job opportunities and income level	Tertiary Industrial Output-Value	RMB
	Urban Residents’ Per Capita Disposable Income	RMB/capita
live condition	Unemployment rate	%
	Participant Rate of Urban Basic Medical Care System	%
	Per Capita Living Area	m^2^/capita
educational opportunities	Number of Regular Primary Schools	unit
	Number of Regular Secondary SchoolsNumber of Universities	unit

Note: RMB is the abbreviation of Ren Min Bi (China Yuan), which is the basic monetary unit of China.

**Table 4:** The Pearson coefficient between population migration and external-migration attention indexe (MAI).

Region	Three UAs	BTH	YRD	PRD
Coefficient	0.844	0.948	0.876	0.879
Sig(2-side)	0.000	0.000	0.000	0.002

Note: UA: urban agglomeration; BTH: Beijing-Tianjin-105 Hebei metropolitan region; YRD: the Yangtze River Delta; PRD: the Pearl River Delta.

**Table 5:** The Pearson coefficient between urban comprehensive attractiveness for migrants (UAM) and external-MAI.

Region	Three UAs	BTH	YRD	PRD
Coefficient	0.829	0.924	0.984	0.789
Sig(2-side)	0.000	0.000	0.000	0.020

Note: UA: urban agglomeration; BTH: Beijing-Tianjin-105 Hebei metropolitan region; YRD: the Yangtze River Delta; PRD: the Pearl River Delta.

**Table 6:** Correlation coefficient between external-MAI and urban pulling indicators.

Perspective	Index	ALL	BTH	YRD	PRD
**job opportunities and income level**	TIV	0.869*	0.913*	0.971*	0.869*
	IPC	0.598*	0.921*	0.744*	0.800*
**live condition**	UR	−0.151	−0.331	0.129	−0.197
	RBM	0.509*	0.916*	0.782*	0.485
	LPC	0.093	−0.203	0.264	0.217
**educational opportunities**	SSN	0.677*	0.851*	0.963*	0.509
	PSN	0.840*	0.865*	0.944*	0.744*
	UN	0.759*	0.930*	0.976*	0.477

Note: *: Pearson correlation is significant at the 0.01 level. TIV: Tertiary Industrial Output-Value; IPC: Urban Residents’ Per Capita Disposable Income; UR: Unemployment Rate; RBM: Participant rate of Urban Basic Medical Care System; LPC: Per Capita Living Area; SSN: Number of Regular Secondary Schools; PSN: Number of Regular Primary Schools; UN: Number of University.

**Table 7:** The Pearson coefficient between local-MAI and floating population.

Region	Three UAs	BTH	YRD	PRD
Coefficient	0.853	0.889	0.950	0.780
Sig(2-side)	0.000	0.000	0.000	0.013

Note: UA: urban agglomeration; BTH: Beijing-Tianjin-105 Hebei metropolitan region; YRD: the Yangtze River Delta; PRD: the Pearl River Delta.

**Table 8:** The Pearson coefficient between intercity-MAI and intercity population flow.

Region	Three UAs	BTH	YRD	PRD
Coefficient	0.5685	0.5283	0.5437	0.6369
Sig(2-side)	0.0000	0.0000	0.0000	0.0000

**Table 9:** The variance (VAR) and coefficient of variation (CV) of different types of MAI.

	VAR	CV
External-MAI	0.001	3.93%
Local-MAI	0.005	8.28%
Intercity-MAI	0.002	8.71%
